# Preparation, Optimization, and Investigation of Naringenin-Loaded Microemulsion for Topical Application

**DOI:** 10.52547/ibj.3722

**Published:** 2022-11-20

**Authors:** Anayatollah Salimi, Sara Amirimoghadam, Farid Bagheri

**Affiliations:** 1 Nanotechnology Research Center, Ahvaz Jundishapur University of Medical Sciences, Ahvaz, Iran;; 2 Department of Pharmaceutics, Faculty of Pharmacy, Ahvaz Jundishapur University of Medical Sciences, Ahvaz, Iran

**Keywords:** Naringenin-Loaded Microemulsion, Topical Application, Treatment

## Abstract

**Background:**

Flavonoids are a large group of phenolic compounds possessing anti-inflammatory and antioxidant effects. NAR is a flavonoid with various pharmacological properties. Using pharmaceutical compounds on skin is one of the routes of administration to achieve local and systemic effects. The aim of this study was to develop a topical formulation of NAR by the preparation of a NAR ME, which was further tested its skin permeability in rats.

**Methods:**

Eight 0.5% NAR MEs were prepared by mixing appropriate amounts of surfactant (Tween 80 and Labrasol), cosurfactant (Capryol 90) and the oil phase (oleic acid-Transcutol P in a ratio of 1:10). The drug was dissolved in the oil phase. The physicochemical properties of MEs such as droplet size, viscosity, release, and skin permeability were assessed using Franz Cells diffusion.

**Results:**

Based on the results, the droplet size of MEs ranged between 5.07 and 35.15 nm, and their viscosity was 164-291 cps. Independent factors exhibited a strong relationship with both permeability and drop size. The permeability findings revealed that the diffusion coefficient of NAR by the ME carrier increased compared to the drug saturation solution.

**Conclusion:**

The most validated results were obtained for Jss and particle size. Optimal formulations containing MEs with Jss and particle sizes varying between minimum and maximum amounts are suitable for topical formulations of NAR.

## INTRODUCTION

Flavonoids are low molecular weight secondary metabolites characterized by a flavonoid structure. More than 4,000 flavonoids have been widely identified in the leaves, seeds, barks, and flowers of the plants, which protect them against UV rays, pathogens, and vegetarians ^[^^1^^,^^2^^]^. Flavonoids are a group of natural substances with variable phenolic structures. These compounds are rich in antioxidant activity and can help body rid itself of daily toxins ^[^^1^^,^^2^^]^.

 NAR (4,5,7-trihydroxyflavanone) is a citrus flavanone (a subgroup of flavonoids) with a molecular weight of 272.26 and abundantly found in fruits such as grape, grapefruit, blood orange, lemons, pomelo, and tangerine^[^^3^^,^^4^^]^. NAR has a broad variety of biological properties for human health. Its antioxidant activity, as well as ability to chelate metals, scavenge oxygen free radicals, inhibit enzymes, and prevent oxidation of low-density lipoproteins are the primary reasons for its administration^[^^5^^,^^6^^]^. NAR is a hydrophilic compound with high solubility in water, which makes it easy to use for therapeutic applications, especially topical treatments^[^^7^^]^. Skin is the body's first layer of defense; it acts as a barrier to the flowing of foreign substances such as drugs into the body. Among different skin layers, the stratum corneum plays the most important role in contributing to defense system^[^^8^^]^. 

To date, various new strategies have been proposed to improve the penetration of drug into the skin and increase percutaneous absorption, which temporarily destructs stratum corneum. ME carriers are one of these strategies that thermodynamically stabilize dispersion of oil, water, surfactant, and often cosurfactant. Surfactants or lipids used in MEs dissolve the lipid bilayer structure of the stratum corneum, thereby creating pores in the skin that help the drug penetration into the skin^[^^9^^]^.

Topical preparations containing NAR (0.5%) have been reported to exert anti-inflammatory and antioxidant properties and protect skin against UVB damage^[^^10^^]^. Considering the above outcomes, this study attempted to prepare a topical form of NAR by first preparing a NAR ME (5%) and then testing the skin permeability of this compound in rats.

## MATERIALS AND METHODS

**Materials**

NAR powder was procured from Sigma-Aldrich (USA). ME carrier components, including caprylo- caproylmacrogoglycerides (Labrasol), propylene glycol monocaprylate (Capryol 90) and diethylene glycol monoethyl ether (Transcutol P), were purchased from Gattefosse Co. (France). Oleic acid and Tween 80 were acquired from Merck Co. (Germany). 

**Animals**

Male Wistar rats weighing 150-170 g and aged 10-12 weeks were used in this study. All the rats were anesthetized by ketamine (80 mg/kg)/xylazine (10 mg/kg) as per the guidelines codified by the Ethics Committee of Ahvaz Jundishapur University of Medical Sciences (Ahvaz, Iran). The hairs on the rats' abdomens were shaved, and the whole skin of the abdomen was excised after slaughtering. The skins were maintained in an aluminum foil in a freezer at -20 °C until the test was performed. Thereafter, the subcutaneous fat was removed using pure cold acetone. 

**NAR solubility**

The solubility of NAR in oleic acid, oleic acid + Transcutol P (10:1), surfactants (Tween 80, Labrasol), cosurfactant (Capryol 90), and water was measured by dissolving an excess amount of NAR in 5 ml of the aforementioned supernatant. The samples were mechanically mixed at 25 ± 0.5 °C for 72 h. After equilibration, the transparent supernatants were filtered through a polytetrafluoroethylene membrane filter (0.45 μm), and then the filtrates were examined by a UV spectrophotometer at 325-nm wavelength^[^^11^^]^.

**NAR determination**

A UV spectrophotometer with a wavelength of 325 nm was used to determine the parameters of ME, including drug loading and release. Wavelengths were chosen based on the spectral absorbance of NAR in a 2:1 ratio of phosphate (pH 7.4) and methanol. NAR had no absorbance and showed no interference with other compounds^[^^12^^]^.

**Pseudo-ternary phase diagram construction**

A pseudo-ternary phase diagram was prepared using water titration to distinguish the zones of the formed ME. Based on the 4:1 and 6:1 weight ratios of Labrasol-Tween 80/Capryol 90, two-phase diagrams were plotted. The oil phase (oleic acid + Transcutol P) ratio (10:1) to the mixture of surfactant and cosurfactant (Tween 80-Labrasol/Capryol 90) were designed at the ratios of 1:9, 2:8, 3:7, 4:6, 5:5, 6:4, 7:3, 8:2, and 9:1. The oil phase, surfactant, and cosurfactant combination were titrated dropwise by deionized water and agitation. The translucent and homogeneous samples were considered to represent ME zones in the phase diagram after equilibration^[^^13^^]^.

**Preparation of ME**

**Fig. 1:**
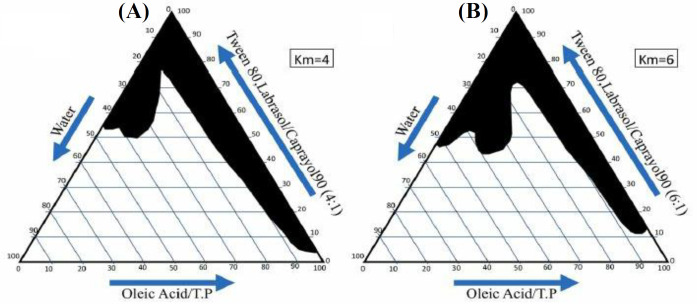
The pseudo-ternary phase diagrams of the oil-s/c mixture-water system ambient temperature. Dark areas show ME zone

To prepare MEs, we used pseudo-ternary phase diagrams (Fig. 1). In brief, eight formulations were prepared using full factorial design with three variables at two high and low levels (Table 1). The independent variables (0.5%) consisted of the ratio of s/c, oil, and water content. In a test tube, NAR was introduced to the oil phase, followed by the s/c combination in two phases, and finally water was added dropwise. Vortex and sonicator were used at all steps to mix the components and obtain a transparent NAR ME^[^^14^^]^.

**Determination of ME droplet size**

The mean droplet size and dispersion index of MEs were measured using the particle size analyzer (Agilent, USA). The particle size distribution, including average droplet size of samples, was measured by SCATTER SCOPE 1 QUIDIX (South Korea) at 25 °C, and the refractory indices were calculated. Subsequently, samples were separately transferred to a special tube and examined against laser radiation.

**Evaluation of viscosity and pH of drug-containing MEs**

The rheological properties of MEs were evaluated by a viscometer DV-II + Pro (Brookfield, USA) at 25 ± 1 °C and spindled 34 at 75 rpm. Then 10 ml of the sample was used for viscosity measurements. pH of the samples was adjusted by 0.1 N of NaOH using a pH meter (Mettler Toledo Seven Easy, Switzerland). 

**Drug release from ME formulations**

Static Franz diffusion cells with a cross-sectional area of ​​4.906 cm^2^ were used to obtain the drug release profile. As a dialysis membrane, a cellulose membrane (3000-4000 kDa) was put in deionized water 24 hours before the experiment and then clamped between the donor and receptor chambers of the diffusion cells. Thereafter, two mg of each ME was placed on the membrane, and the receptor chamber was filled with a mixture of pH (4-7) methanol at the ratio of 2:1, and then a magnet was placed inside deionized water. The cells were then placed on a steering device at 200 rpm, and the receptor medium was set at 37 ± 0.5 °C. At the specified intervals (0.5, 1, 2, 3, 4, 5, 6, 7, 8, and 24 h), 2 ml of the receptor phase was removed and replaced by 2 ml of a fresh blank medium. The amount of the drug dissolved in the receptor phase was determined using spectroscopy at the wavelength of 325 nm. The experiment was carried out in triplicate for each sample^[^^14^^]^.

**Evaluation of NAR skin permeability**

Rats’ abdominal skin were cut into tiny pieces and clamped between the donor and receptor chambers of the Franz diffusion cells such that the stratum corneum faced the donor chamber. After being taken out of the freezer to reach room temperature, the skin was hydrated between two chambers, and the phases were then drained and dried on the skin. Next, 3 mg of the drug was introduced into the donor chamber, and 35 ml of methanol buffer, along with a magnet, was placed in the receptor chamber. The receptor medium was set at 37 ± 0.5 °C. At 0.5, 1, 2, 3, 8, 24, 26, 28, 32, and 48 h intervals, 2 ml of the receiver chamber was removed, and 2 ml of the fresh blank media was replaced. The amount of drug dissolved in the receptor phase was measured by UV spectroscopy at 325-nm wavelength. In this experiment, the saturated solution of NAR was used as the control^[^^8^^]^.

**Data analysis and calculation of permeability parameters**

Studen’s t-test and analysis of variance (ANOVA) were used to conduct data analysis. Minitab 17 software was also used to design the full-factorial experiment. The results were plotted as cumulative permeated drug percentages versus time. Based on the plots, permeability parameters, such as Jss, permeability coefficient (P), lag time (t_lag_), and apparent diffusion coefficient (D_app_) were calculated. The permeability coefficient (P) was calculated using the equation J_SS_ = P × C^[^^15^^]^, where C is drug concentrati on in the donor phase. The value of D was determined using equation D = $\frac{h^{2}}{6_{\mathrm{tlag}}}$^[^^16^^]^. As h is not the real duration of drug transit, D_app_ may be calculated using this method. 

**Table 1:** Composition of the prepared NAR MEs

**Water (%)**	**s + c (%)**		**s/c**	**Factorial design**	**Formulation**
30	60	10	6:1	+ + +	ME-NAR-1
20	70	10	6:1	- + +	ME-NA--2
30	65	5	6:1	+ - +	ME-NAR-3
20	75	5	6:1	- - +	ME-NAR-4
20	75	5	4:1	+ - -	ME-NAR-5
30	65	5	4:1	- - -	ME-NAR-6
20	70	10	4:1	- + -	ME-NAR-7
30	60	10	4:1	+ + -	ME-NAR-8

**Table 2:** Solubility of NAR in different oils, surfactant, and cosurfactants

**Phase type**	**Excipient**	**Solubility (mg/mL)**
Oil	Oleic acid	12.5 ± 0.1
	Transcutol P	375 ± 2.0
		
Surfactant	Tween 80	83.5 ± 1.5
	Labrasol	131 ± 1.2
		
Cosurfactant	Capriol 90	127 ± 1.5
Water		0.042 ± 0.005
Oleic acid + Trancutol P		65 ± 0.008

Data were presented as the mean ± SD of triplicate experiments.

Because all computations were predicated based on the steady-state portion of the cumulative drug permeability diagram, synchronized criteria were specified for the validity of these values. The greatest concentration generated in the receptor phase was less than 10 times that of the drug saturation solution in our study.

## RESULTS AND DISCUSSION

**NAR solubility results**

**Fig 2:**
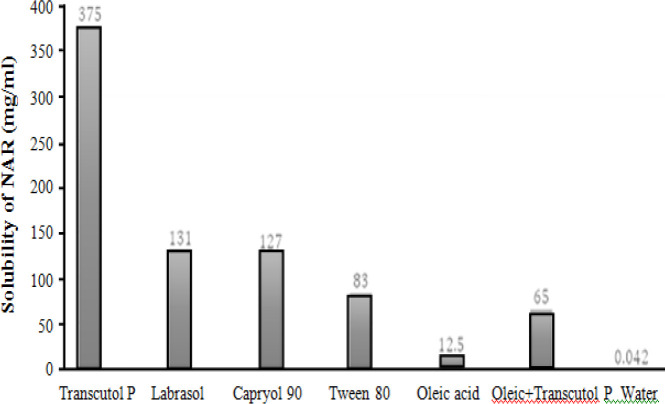
Solubility profile of NAR in different components of ME

Solubility of NAR is shown in Table 2 and Figure 2.

**Properties of NAR MEs**

***Viscosity, particle size, and pH of MEs***

**Fig. 3:**
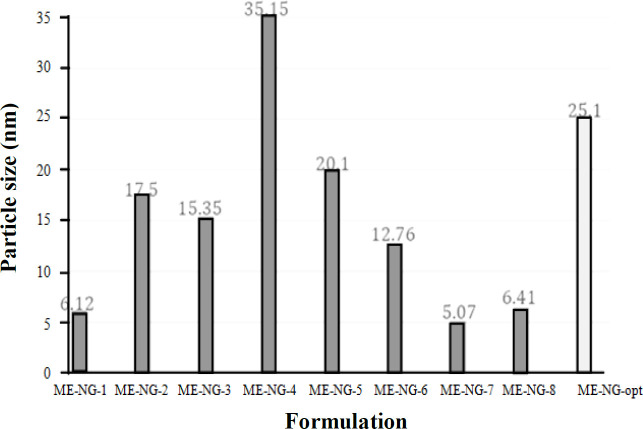
Particle size of optimal formulation in comparison with other formulations

According to our results, ME-7 and ME-4 had the lowest and highest particle size, respectively (Table 3, Fig. 3). The droplet size distribution is one of the most important features affecting the *in vivo* fate of MEs. This feature can determine the amount and rate of drug release^[^^17^^]^. The mean droplet size, calculated in this study, was less than 100 nm. The droplet size range was between 5.07 to 35.15 nm. The particle dispersionindex of MEs, which indicates the uniformity of particle size was less than 0.5 nm (Table 3). The average droplet size had a substantial and inverse correlation with the proportion of water and oil (Fig. 3); however, it had a significant direct correlation with the s/c ratio, which are in line with the studies conducted by Salimi *et al.*^[^^14^^]^ and Censi *et al.*^[^^18^^]^. Furthermore, ME-8 and ME-5 had the lowest and highest viscosity, respectively, at 75-rpm shearing rate (164 vs. 291cps; Table 3). Viscosity of the ME is an important factor for drug penetration into the skin and the formulation stability which widely depends on its constituents. In this context, it is well known that various kinds of micelles may be generated by raising the surfactant content, which increases the total viscosity^[^^18^^]^. Moghimipour *et al*.^[^^11^^]^ reported similar results for viscosity of tretinoin ME. The pH of MEs is 3.94-4.55. It has been reported that the pH of azelaic acid ME with similar components to NAR ME was also reported to be approximately 4.5^[^^13^^]^. Our data analysis showed a significant correlation between pH of ME and water percentage as well as s/c ratio. There was an inverse correlation between pH and water and a direct association between pH and the s/c ratio.

**Table 3:** The pH, mean droplet size, polydispersity index, and viscosity of NAR MEs

**Viscosity (cps)**	**Polydispersity index**	**Mean droplet size (nm)**	**pH**	**Formulation**
261 ± 1.4	0.353 ± 0.007	6.12 ± 0.71	4.03 ± 0.02	ME-NAR-1
206 ± 6.3	0.389 ± 0.004	17.50 ± 0.61	4.33 ± 0.02	ME-NAR-2
204 ± 4.8	0.334 ± 0.004	15.35 ± 0.64	3.98 ± 0.06	ME-NAR-3
268 ± 3.9	0.316 ± 0.001	35.15 ± 0.92	4.55 ± 0.02	ME-NAR-4
291 ± 2.4	0.316 ± 0.001	0.10 ± 0.95	4.28 ± 0.02	ME-NAR-5
261 ± 2.8	0.353 ± 0.004	12.76 ± 0.57	3.94 ± 0.11	ME-NAR-6
224 ± 3.4	0.334 ± 0.011	5.07 ± 0.06	4.22 ± 0.02	ME-NAR-7
164 ± 5.2	0.316 ± 0.006	6.41 ± 0.07	3.89 ± 0.02	ME-NAR-8

Data were presented as the mean ± SD of triplicate experiments.

**Profile of NAR release**

**Fig. 4:**
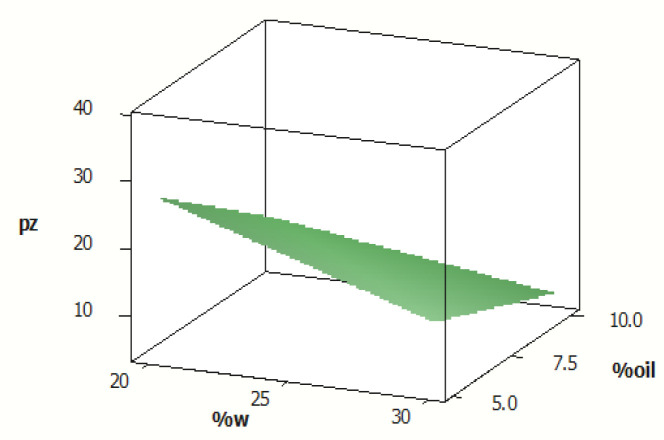
The surface diagram of droplet size (nm), water (w) percentage, and oil percentage. Pz, particle size

**Fig. 5:**
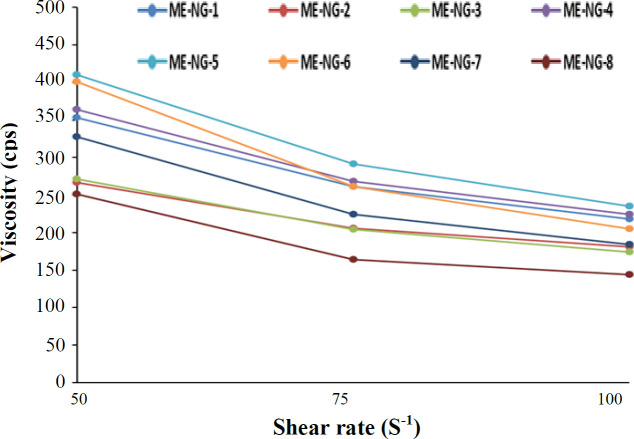
Rheogram viscosity (cps) versus the shear rate of MEs at 25 °C (mean ± SD of triplicate experiments)

The highest and lowest amounts of drug release were observed at 4-h and 24-h intervals for ME-2 and ME-7, respectively (Table 4). The diagram of cumulative release of NAR MEs is illustrated in Figures 4 and 5. Percentage of drug release is one of the crucial properties of the formulation that plays a key role in 

exerting therapeutic effect. Our findings showed no significant association between the percentages of drug release at 4-h and 24-hour intervals and independent factors. Except for formulation 2, kinetics of drug release from formulations follows Higuchi's model, which indicates that drug release is controlled by diffusion^[^^19^^]^.

***In vitro*** **permeation studies**

Data for the permeability parameters of NAR MEs and their comparison with the control of saturation of drug solution on the abdominal skin of rat are shown in Tables 5 and 6. Data analysis revealed a significant correlation between Jss, or D, and t_lag_ with the s/c ratio in which its association with D was direct, but with Jss and t_lag_ was inverse. Increased permeability or drug disposition in the skin can occur due to various reasons, including type of ME components and ability of these drugs to enhance penetration, the surface tension of the formulation, the contact surface, and the ability of ME components to dissolve drug^[^^19^^]^. As mentioned above, the type of ME component is a factor drug in the skin. Transcutaneous P was one of the components used in the current study. The hygroscopic liquid Transcutaneous P is miscible with both polar and non-polar solvents. Transcutol P is a nontoxic, skin biocompatible absorption booster with excellent effect on improving NAR and quercetin solubility. In a study conducted on the preparation of quercetin ME, Transcutol P was used in the formulation, which could help to dissolve and penetrate quercetin through the skin, though it was not suitable penetrated. Quercetin was completely dissolved in Transcutol P and into the skin after the release of excipient^[^^20^^]^. Oleic acid is an unsaturated fatty acid with a cis spatial configuration and used as the oil phase , making it a useful absorption enhancer. Due to the uneven arrangement of the lipid chains in the intercellular bilayer structure, oleic acid causes irregularity and inhibits the temperature transfer of the gel phase to the liquid crystal after passing through the skin^[^^21^^]^. Oleic acid improves penetration via lipid disruption, which is thought to be occurred through two mechanisms: conformational permutation and phase separation^[^^21^^]^. Surfactants increase drug permeation through dissolving stratum corneum lipids^[^^22^^]^. Overall, most enhancers interact with the intercellular lipid domain of the stratum corneum^[^^22^^]^. Herbal active ingredients in the form of carriers and drugs can pass through the membrane and therefore be used for medicinal and therapeutic purposes^[^^23^^-^^28^^]^. ME enhances partition coefficient and flux of NAR, but not NAR-saturated solution. Enhanced flux and partition coefficient of NAR MEs may be due to the fact that oleic acid and surfactants liquefy the lipid matrix or destroy the lipid structure of the stratum corneum. The results showed that all formulations, except for formulations 7 and 8, raised diffusion coefficient compared to the NAR saturated solution. The target value of the droplet size and flux of the optimal formulation determined that the Jss parameter was minimum and maximum at 0.0005 ± 0.00465 and 0.0001 ± 0.0278, and the particle size parameter was minimum and maximum at 0.06 ± 5.07 and 0.92 ± 35.15, respectively. The percentage and ratio of the optimal formulation were also 7.0482% (oil), 20.0631% (water), and 4.5185 (s/c). 

**Table 4:** Data on drug release of ME formulations

**R**_24 h_ **(%)**	**R**_4 h_ **(%)**	**r** ^2^	**Kinetic**	**Formulation**
41.671 ± 0.845	6.390 ± 0.087	0.8975	Higuchi	ME-NAR-1
61.039 ± 0.518	7.189 ± 0.211	0.8910	First	ME-NAR-2
37.775 ± 0.192	5.953 ± 0.059	0.8900	Higuchi	ME-NAR-3
33.655 ± 0.334	6.077 ± 0.170	0.9109	Higuchi	ME-NAR-4
31.088 ± 0.047	5.483 ± 0.041	0.9145	Higuchi	ME-NAR-5
40.063 ± 0.016	6.62 ± 0.015	0.8901	Higuchi	ME-NAR-6
30.898 ± 0.314	5.062 ± 0.510	0.9206	Higuchi	ME-NAR-7
40.470 ± 0.510	6.371 ± 0.036	0.8907	Higuchi	ME-NAR-8

Data are presented as the mean ± SD (m = 3). R_4 h_, release at 4 h; R_24 h_, release at 24 h

**Table 5:** The permeability parameters of NAR ME formulations in comparison with the control of drug saturation

**Formulation**	**Jss (mg/cm** ^2^ **/h)**	**D**_app _ **(cm** ^2^ **/h)**	**P (cm/h)**	**t**_lag _ **(h)**	**Q**_24_
ME-NAR-1	0.0104 ± 0.0003	0.053 ± 0.013	0.0113 ± 0.013	2.59 ± 0.595	0.039 ± 0.05
ME-NAR-2	0.00456 ± 0.0005	0.429 ± 0.077	0.0009 ± 0.077	0.319 ± 0.057	0.162
ME-NAR-3	0.0102 ± 0.0003	0.065 ± 0.0026	0.0038 ± 0.002	1.64 ± 0.065	0.212
ME-NAR-4	0.0113 ± 0.0004	0.094 ± 0.005	0.0023 ± 0.49	1.131 ± 0.057	0.1949
ME-NAR-5	0.009 ± 0.0034	0.0326 ± 0.012	0.0012 ± 0.015	3.866 ± 0.853	0.07
ME-NAR-6	0.0084 ± 0.0012	0.0430 ± 0.027	0.0017 ± 0.0002	3.332 ± 1.620	0.082
ME-NAR-7	0.0278 ± 0.0001	0.023 ± 0.0001	0.005 ± 0.001	4.55 ± 0.022	0.18
ME-NAR-8	0.0210 ± 0.0700	0.026 ± 0.004	0.004 ± 1.41	4.707 ± 0.024	0.12
Control	0.002 ± 0.0003	0.0263 ± 0.002	0.0004 ± 0.071	4.067 ± 0.318	0.016 ± 0.0003

Data are presented as the mean ± SD of triplicate experiments. Jss, steady-state flux; D_app_, apparent diffusion coefficient; P, permeability coefficient; t_lag_, lag time

**Table 6:** The permeability parameters of NAR MEs in comparison with NAR-saturated solution

**Formulation**	**ER**_D_	**ER**_P_	**ER**_flux_
ME-NAR-1	2.016 ± 0.307	24.066 ± 26.069	4.94 ± 0.977
ME-NAR-2	16.485 ± 0.593	2.212 ± 0.594	2.212 ± 0.594
ME-NAR-3	2.468 ± 0.095	9.249 ± 0.207	4.798 ± 0.657
ME-NAR-4	3.605 ± 0.464	5.344 ± 1.076	5.344 ± 1.076
ME-NA -5	1.225 ± 0.375	31.375 ± 4.481	4.375 ± 2.289
ME-NAR-6	1.600 ± 0.903	3.937 ± 0.088	3.937 ± 0.088
ME-NAR-7	0.893 ± 0.065	11.0186 ± 1.035	13.102 ± 2.089
ME-NAR-8	0.972 ± 0.090	9.922 ± 1.599	9.922 ± 1.598

Data were presented as the mean ± SD of triplicate experiments.

**Table 7:** Permeability parameters from the optimal formulation of NAR

**Formulation**	**t**_lag _ **(h)**	**P (cm/h)**	**D**_app _ **(cm** ^2^ **/h)**	**Jss (mg/cm** ^2^ **/h)**	**ER**_P_	**ER**_D_	**ER**_flux_
ME-NAR-opt	1.921±0.027	0.014±0.017	0.070±0.001	0.013±0.0002	31.287±2.124	2.68±0.24	6.35±1.14

tlag, lag time; P, permeability coefficient; Dapp, apparent diffusion coefficient; Jss, steady-state flux

**Fig. 6:**
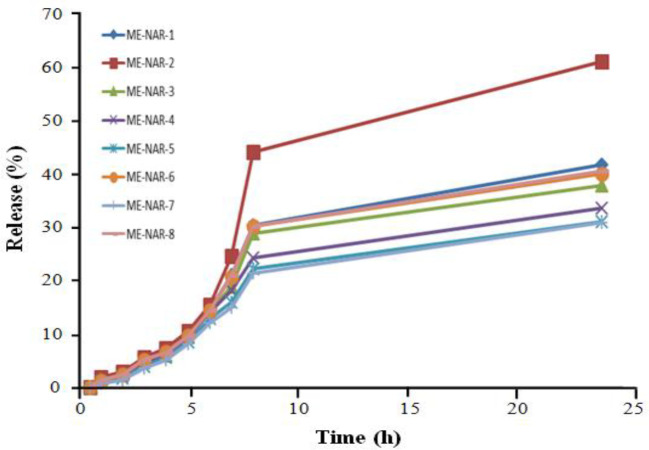
Cumulative release graph of NAR ME formulations

**Optimal formulation**

**Fig. 7:**
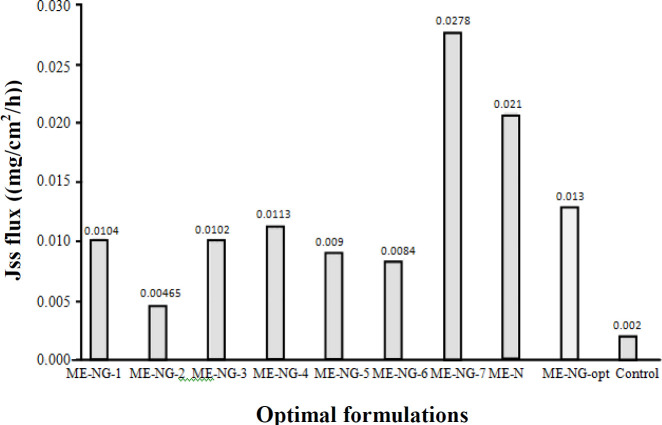
The Jss flux of optimal formulation in comparison with other formulations

The most optimum formulation was developed based on the droplet size and flow data (as the most validated data). The target values of the droplet size and the flux of the optimal formulation were determined out of the lowest and highest data. The most appropriate proportion of phases in which the droplet size and the flux of the drug were equal to our target values was introduced as the optimal formulation by targeting those values. The results of permeability parameters of the optimal formulation of NAR in comparison with the control of drug saturation solution are shown in Table 7. The particle size of optimal formulation is 25.1 ± 1.4. The Jss and particle size of the optimal formulation range the maximum and minimum of these variants. Optimal formulation can be prepared using a suitable amount of NAR in epidermal layers, so that such formulation can be used to prepare a topical form of NAR (Figs. 6 and 7). 

In conclusion, the results for Jss and particle size were significant and important. The formulation optimized for this study with Jss and MEs particle size is suitable for the topical formulation of NAR.

## DECLARATIONS

### Acknowledgments

The authors gratefully thank the Vice Chancellor for Financial support and use of devices from Research and Technology of the University. The authors are also thankful to Iranian Representation for Gattefosse Pharmaceuticals (Faratin Company).

### Ethical statement

The study protocol was approved by the Ethics Committee of the Ahvaz Jundishapur University of Medical Sciences, Ahvaz, Iran (ethical code: IR.AJUMS.ABHC.REC.1399.064). 

### Data availability

 The analyzed data sets generated during the study are available from the corresponding author on reasonable request.

### Author contributions

**AS: data analysis; SA: manuscript scanning; FB: data evaluation. All authors have read and approved the final version of manuscript.**

### Conflict of interest

None declared.

### Funding/support

This study was funded by Ahvaz Jundishapur University of Medical Sciences, Ahvaz, Iran.
